# The Pay of Medical Officers

**Published:** 1923-09

**Authors:** 


### THE MEDICAL OFFICER'S PAY.

It is not difficult to understand that a certain number of Councils are somewhat alarmed at the new scale of salaries for Medical Officers of Health which has just been adopted by the British Medical Association. When a small town is faced by an ultimatum from a powerful professional trade union, prohibiting it from appointing a whole time Medical Officer at a lower salary than £800, a sense of grievance is not unnatural. Most of them have hitherto paid on a much lower scale, and they may be expected to ask why a community of, say, 30,000 people should be called upon to give a salary of that amount when a great city with nearly half a million population pays only double. But to men of experience in local administration the answer is fairly obvious. As a rule the minimum salary man works single-handed, whereas the principal health officer of a great town is provided with various departmental assistants who, if they cannot relieve him of responsibility, can, and do, relieve him of a great deal of actual labour.

There have already, as at St. Pancras and Walsall, been one or two conflicts between the scale and the Councils, and there may yet be others, but we have little doubt that a general understanding will soon be reached. To accuse the British Medical Association or the Society of Medical Officers of Health of being grasping tyrants, seeking to force an extravagant scale upon hardly-pressed spending authorities, is absurd. It is beyond doubt that during the post-war period the medical officer has, as often as not, been underpaid, and it was time that his remuneration should be placed on a reasonable footing, graduated in fair relation to his work. This is no more than to say that the labourer is worthy of his hire; but there is another, and a very important consideration, which needs to be pressed upon local authorities, especially those which have not yet quite fully realised that care for the public health is the most onerous of their many duties. The medical supervision of the public health has now become a definite career, calling not only for special acquirements, but for distinct formal qualifications, as the article on the subject which we print on another page makes abundantly clear. The aspirant to the public health service must not only be fully qualified in the ordinary sense of the word, but it is becoming the rule that he must also possess the D.P.H., which is a training in itself, and must have acquired experience in tuberculosis, school hygiene, or some other specialised work. It cannot be pretended that a young scientific man, who has spent several years in fitting himself for a career in which the public health will, in no small degree, be at his mercy, is over-paid with a commencing salary, as a junior officer, of £600 a year.

That the public health service should now have become a career needing special aptitudes and qualifications, continuing study, and persistent keeping abreast of new discoveries and the advance of sanitary science, is cause for very real satisfaction. The old happy-go-lucky days have gone by and the proper care of the health of a community of any size is now clearly seen to require the unremitting attention of an officer whose attainments have especially adapted him for the work.

